# The power of negative and positive episodic memories

**DOI:** 10.3758/s13415-022-01013-z

**Published:** 2022-06-14

**Authors:** Samantha E. Williams, Jaclyn H. Ford, Elizabeth A. Kensinger

**Affiliations:** Department of Psychology and Neuroscience, Boston College, McGuinn Hall, Rm 300, 140 Commonwealth Ave., Chestnut Hill, MA 02467 USA

**Keywords:** Emotional memory, Valence, Encoding, Retrieval

## Abstract

The power of episodic memories is that they bring a past moment into the present, providing opportunities for us to recall details of the experiences, reframe or update the memory, and use the retrieved information to guide our decisions. In these regards, negative and positive memories can be especially powerful: Life’s highs and lows are disproportionately represented in memory, and when they are retrieved, they often impact our current mood and thoughts and influence various forms of behavior. Research rooted in neuroscience and cognitive psychology has historically focused on memory for negative emotional content. Yet the study of autobiographical memories has highlighted the importance of positive emotional memories, and more recently, cognitive neuroscience methods have begun to clarify why positive memories may show powerful relations to mental wellbeing. Here, we review the models that have been proposed to explain why emotional memories are long-lasting (durable) and likely to be retrieved (accessible), describing how in overlapping—but distinctly separable—ways, positive and negative memories can be easier to retrieve, and more likely to influence behavior. We end by identifying potential implications of this literature for broader topics related to mental wellbeing, education, and workplace environments.

Episodic memories are powerful in their ability to transport us back in time, allowing us to reexperience and reflect on past moments (Tulving, 1972). As eloquently described by the concept of the “episodic buffer” (Baddeley, 2000), when we bring an episodic memory to mind, we bring the past into the present—into the current content of our consciousness. By doing so, we use the past to guide our current decisions and to shape our predictions about future occurrences (Gershman, 2017).

Most people have the intuition that emotional experiences—those that get our heart racing or that elicit a positive or negative reaction—are more likely to be retained in memory, and the data support this conjecture. While we do not retain all the details of an emotional experience accurately (Neisser & Harsch, 1992), we are less likely to altogether forget that it occurred (Kensinger & Schacter, 2008; Yonelinas & Ritchey, 2015). Meanwhile, many of life’s more mundane moments crumble from our memory stores, losing the ability to influence our behavior or predictions.

Emotional experiences also can be more accessible in memory than neutral experiences (Buchanan, 2007; van Schie et al., 2015). We may have to search effortfully to recall the last birthday of an acquaintance, whereas the details of a past emotional event may spring to mind effortlessly as we approach its anniversary. Thus, the collection of episodic memories available for us to bring into present consciousness will be biased toward the highs and lows that we have experienced, giving them more influence over our current decisions and predictions for the future.

We first review the evidence for the power of negative memories and then for the power of positive memories. We explain how emotion evokes mechanisms that lead these experiences to be retained in memory and accessed at retrieval, and we describe some of the consequences that the durability of these emotional memories have on our decisions, behaviors, and wellbeing. We end by pointing out potential implications of this literature for broader topics related to mental wellbeing, education, and workplace environments.

## A Note on Terminology

Before launching into this review, we want to note some common confusions that can arise when using the term “emotional memory.” We use this term to describe how the emotion *during an initial experience* affects episodic memory. Although this is a common use of the term, there are several other ways that people might use or interpret the phrase “emotional memory” (see Box 1). Throughout this article, we will use “emotional memory,” and its branches of “negative memory” and “positive memory,” to refer to episodic memories for events that initially elicited a negative or positive affective response.

**Table:** 

**Box 1. Clarifying terminology** We define emotional memory as memory *for a past event that elicited an emotional response.* This definition should not be confused with other possible meanings of “emotional memory”: - *The emotion of the memory*. The use of “emotional” as a modifier to “memory” might suggest that it is the emotion *of the memory* that is being described*.* Yet as we use the term, it is entirely possible to have an “emotional memory” with episodic content, but little emotion reexperienced at the time the memory is brought to mind. - *Memory for a past emotional state.* “Emotional memory” also could refer to memory *of a past emotion,* with the individual trying to remember how they felt previously. But in most studies of “emotional memory,” what is being queried is not memory for the *emotion*, but rather memory for the *experience* that triggered the earlier emotional response. Furthermore, extensive research has shown that people are quite bad at remembering a past emotional (or other mental) state; there can be disconnects between the emotional intensity experienced at encoding and retrieval (Hutchison et al., 2021; Levine et al., 2020), and the emotional state we remember has as much to do with the state we are currently in as with the state we previously experienced (Chang et al., 2018; Levine, 1997). - *Modulation of memory by mood or stress*. Sometimes, “emotional memory” can encompass the study of how a person’s emotional state—the mood they are in or their stress level—influences memory. We do not specifically delve into these influences here, although in some studies it is ambiguous whether effects are driven only by short-lived emotional reactions or by longer-term changes in a person’s state. - *Memory for events that triggered feelings.* When many affective scientists use the term “emotion,” they are talking about states associated with some conscious feeling and often are referring to feeling-states that we name (happiness, sadness, etc.). While there are some exceptions (Riegel et al., 2022), the bulk of the studies on “emotional memory” do not focus on discrete emotions (e.g., distinguishing fear memories from disgust memories). Moreover, many who study “emotional memory,” including ourselves, do not assume that participants are experiencing consciously accessible feeling states in all paradigms (such as when we use stimuli-like words or photo-objects that are only seen for a few seconds). Given the way terms, such as “emotion” and “affect” are used in much current-day discussion (Barrett & Bliss-Moreau, 2009), “memory for affective experiences”—while a mouthful—might be a more accurate summary of what the bulk of research on “emotional memory” has studied. Nevertheless, we will stick with the more commonly used term “emotional memory,” and its derivatives “negative memory” and “positive memory,” to refer to memories for events that, at the time of their occurrence, elicited a negative or positive affective response.	

## Evidence for the Power of Negative Episodic Memories

The study of emotional memories has, until recent years, been dominated by the study of negative memory. It is still the case that many papers whose titles describe a study of “emotional memory” are specifically studying how individuals remember negative content. We glean this focus on the negative to have occurred for two primary reasons.

First, there is a clear power to negative memories and to negative emotions more generally. Baumeister aptly titled a 2001 paper, “Bad is stronger than good” (Baumeister et al., 2001). While it remains debated why that is the case (Alves et al., 2017; Lazarus, 2021), the result often is replicated across many domains. As outlined by Baumeister, people typically attend more to negative information than positive and weight losses more than gains. When constrained to the types of stimuli and participant populations traditionally used in psychology experiments, negativity biases in memory are likely to occur (Bebbington et al., 2017; Vaish et al., 2008). Negative memories also may be particularly durable; individuals may retrieve more remote sad memories than happy ones (Williamson et al., 2019). It is important to recognize that part of the reason for the predominance of the bad may be that, at least for the types of experiences that can easily be assessed in experimental settings, the bad tends to be of greater intensity. It is relatively easy to find photographs or to create vignettes that most people will find alarming or distressing. It is harder to find photographs or to create scenarios that people will find intensely positive, and there tends to be more variability in how people respond to positive stimuli. While this can lead to overestimations of the effects conveyed by negative relative to positive emotion, it also means that when experimenters are trying to use stimuli that will maximize the likelihood of revealing effects of emotion on memory, a focus on the negative is a good strategy.

Second, much of the work in humans was undergirded by a robust literature studying memory in rodents. This literature predominantly focused on how the arousal responses triggered by a shock or another short-lived stressor increased the likelihood that those events were remembered. These memory advantages were revealed to be linked to engagement of the amygdala and to the ability for the amygdala to modulate other medial temporal-lobe and sensory cortical regions (McGaugh, 2000; McGaugh, 2004). Although the amygdala had originally been linked specifically to fear responses (LeDoux, 2003) and to unpleasant stimuli (Lane et al., 1997; Morris et al., 1996), it soon became clear that the amygdala responded to positive as well as negative stimuli (Sergerie et al., 2008) and that memory enhancements extended to pleasurable events as well as aversive ones (McIntyre & Roozendaal, 2007). Despite the advances in the way amygdala reactivity was understood, the connection of these memory effects to arousal responses—and the easier ability to find aversive stimuli that elicit such arousal—likely kept the literature focused on memory for negative experiences. So, that is where we will begin our discussion of the power of emotional episodic memories.

## What Gives Negative Episodic Memories Their Power?

**Fig. 1:**
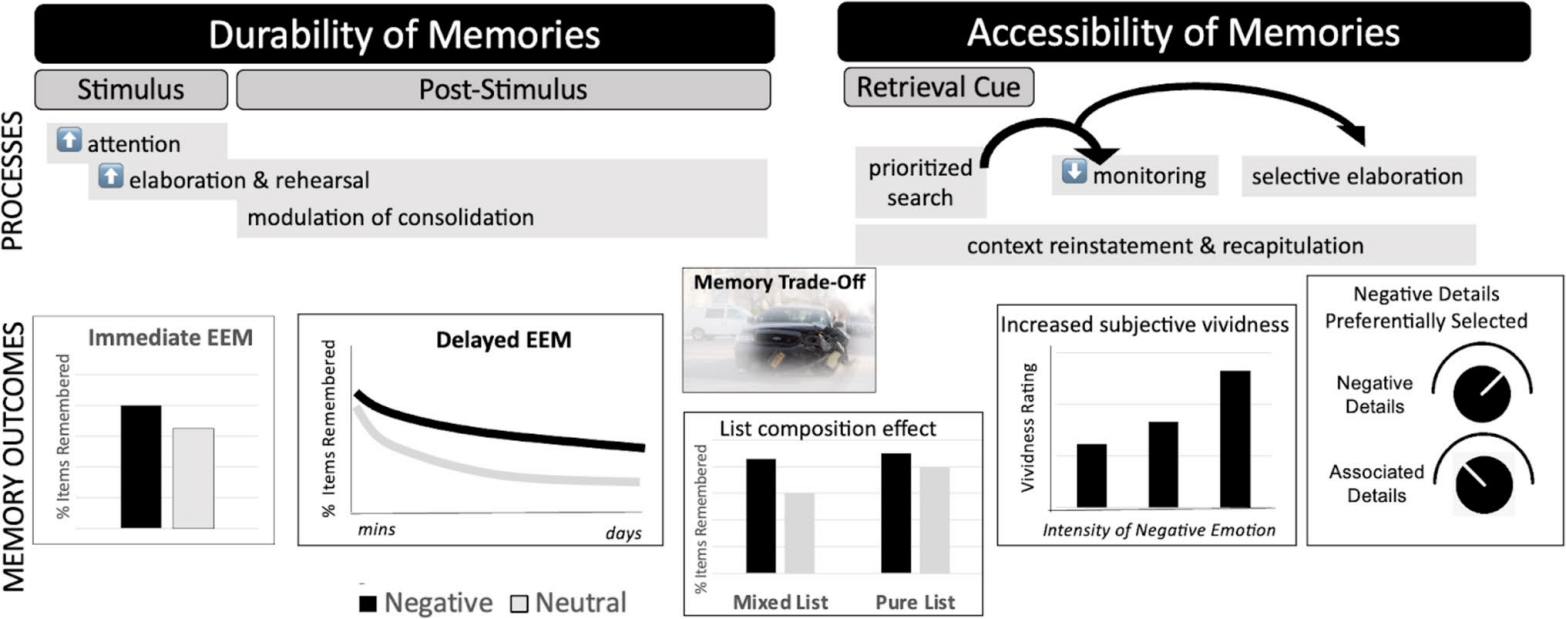
Power of Negative Memories. Processes that unfold during the experience of a negative event, and in the seconds, minutes, and hours that follow, can lead these memories to be durable. Emotional enhancements of memory (EEM) can occur when memory is tested after only a short delay (immediate EEM), and these enhancements can grow as the delay interval increases (delayed EEM). Processes that unfold at retrieval also can increase the likelihood that a retrieval cue brings a negative memory to mind and that the memory is subjectively vivid. Often, what is prioritized at retrieval are the negative details of an event, while the associated contextual details may not be brought to mind. Of course, the processes that unfold at each phase of memory interact with one another, and some of the selectivity of negative memories, such as the tendency for negative memories to retain some details but not others, or for the EEM to be stronger in mixed-lists than pure-lists, likely reflect the way that processes span across these phases. *All figures show mock data; see text for description of related studies*

An obvious answer to the question of what gives negative memories their power is that these memories stick around in our memory stores. From rodents to humans, and from simplistic stimuli to autobiographical experiences, there is abundant evidence that negative content is more likely to be remembered than neutral content, especially over longer-term durations. That is, negative content has a shallower forgetting curve than neutral content (Yonelinas & Ritchey, 2015 for review). Negative memories also may be powerful, because the cues to that content are prioritized at retrieval, and when these memories return to mind, they feel vivid, and people have confidence in their content. Thus, negative memories are powerful, because they have a strength of encoding and consolidation mechanisms that make them *durable* and because at the time of retrieval, they are *accessible* and *vivid* (Figure 1). We describe these features in more detail.

## Negative Memories are Durable

We tend to retain even mundane experiences in memory for short periods of time. We can remember what we just ate for lunch or who sat next to us in the classroom earlier today. Where negative memories start to more noticeably diverge from memories of the mundane is when we examine memory over longer time-frames. We probably *cannot* remember what we ate for lunch 2 weeks ago or who sat next to us in the classroom on the third day of the semester. However, if we found a hair in our food or if our classmate tripped over our backpack on the way to their desk, memory for those negative experiences is likely to stick around longer.

**Fig. 2:**
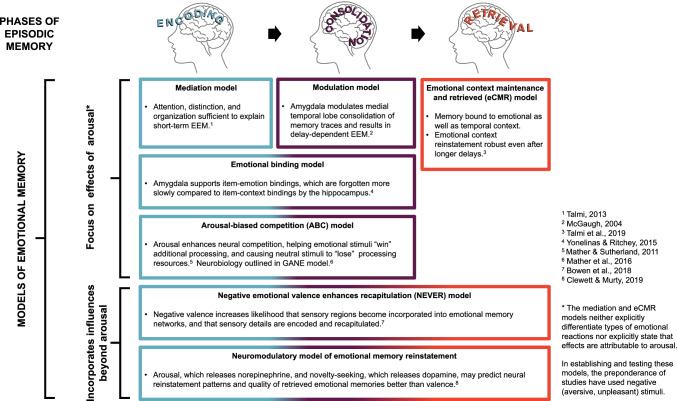
Models of Emotional Memory. There are multiple models of emotional memory. Many are not mutually exclusive, as they focus on different phases of memory or on different characteristics of memory

Many models have been proposed to account for this enhanced durability of emotional memories (see Figure 2 for overview of models of emotional memory). The *modulation model*, developed from studies in rodents, was the first formalized model to explain the emotional enhancement of memory (McGaugh, 2000), and in particular to explain the time-dependency of the enhancement. (We say “formalized model,” because the “now print” mechanism proposed for Flashbulb Memories by Brown & Kulik, 1977 was an influential framework for understanding emotional memories). Extensive research demonstrated that the arousal associated with a (negative) emotional event triggered stress hormones that set off a cascade of processes resulting in upregulation of amygdala function and increasing amygdala-hippocampal connectivity (McGaugh, 2004) and synergy of action (Richter-Levin & Akirav, 2000).

This model emphasized the importance of processes that unfolded during or shortly after an experience to influence the durability of a memory. Not surprisingly, given the influence of this model, the bulk of the initial research trying to understand the durability of humans’ negative episodic memories focused on those phases as well (reviewed by Hamann, 2001). These studies’ results were generally consistent with the *modulation model*: amygdala activity was enhanced during the successful encoding of negative content, and its relation to memory often was related to its interactions with the hippocampus (Richardson et al., 2004).

As additional research was conducted, and as experimental designs were expanded to measure additional aspects of episodic memories, it became clear that the *modulation model* was unlikely to be sufficient to explain the characteristics of negative episodic memories. In particular, the *modulation model* appeared insufficient in describing two key aspects of emotional memories: their tendency to show selective memory enhancements (see also Box 2), and the ability for there to be short-term enhancements in memory before consolidation processes had sufficient time to unfold.

**Table:** 

**Box 2. Models of Selective Memory Enhancements.** There has been longstanding interest in the “memory trade-offs” that occur for emotional memories. Loftus et al. (1987) noted the “weapon focus effect,” whereby individuals remember a weapon but not details of the perpetrator or broader context. Similarly, Reisberg and Heuer (2007) described “emotional memory narrowing,” and Safer et al. (1998) discussed “tunnel memory.” Adolphs et al. (2001) described how emotion seemed to enhance the gist for what had happened but to impair memory for details, and Kensinger and colleagues (Kensinger et al., 2005; Kensinger & Schacter, 2006a) described how emotion can lead central details to be remembered at the expense of their peripheral context. The Arousal Biased Competition (ABC) theory (Mather & Sutherland, 2011) rooted these findings in the biased competition literature (Desimone & Duncan, 1995). Biased competition models essentially propose that there is a tug-of-war for attentional resources, with high-priority stimuli winning and low-priority stimuli losing. ABC suggests that in the presence of arousal, there is an amplification of this tug-of-war, such that the high-priority stimuli take even more of the resources and the low-priority stimuli are left with even less. Support for this model has come from behavioral studies, showing that when a shock or other arousing stimulus is presented, it leads to a greater discrepancy in processing and in memory for the high-priority stimuli (Sutherland & Mather, 2012). Additionally, a neuroimaging study showed that relative to a CS- control tone, when a CS+ tone (predicting a shock) was played, there was both enhanced visual activity for a high-priority visual stimulus and also reduced activity for the low-priority stimulus (Lee et al., 2014). These results suggest that arousal does not uniformly enhance perceptual processing but may do so specifically for high-priority content (see also Clewett & Murty, 2019). It has more recently been proposed, and formalized in the Glutamate Amplifies Noradrenergic Effects (GANE) model, that the neurobiological mechanism underlying this arousal-biased competition may be that “hot spots” are created by synergies between norepinephrine and glutamate release (Mather et al., 2016).	

### Durability for select aspects of negative experiences

Episodic memories are defined by the presence of contextual elements; this context is what makes these memories for *events (episodes)* rather than semantic memories for content void of any context. The multidimensional nature of that context means there are emotional parts to the experience and a myriad of other contextual features that are inconsequential to the emotional experience. Given the role of the hippocampus in binding many of those contextual details together into a stable representation (for different frameworks for this binding, see Backus et al., 2016; Moses & Ryan, 2006; Yonelinas et al., 2019), the *modulation model* might lead to the prediction that negative events should be remembered with a robust array of details. Yet the data have not borne out this prediction (Bisby & Burgess, 2014; Mather, 2016; Sutherland & Mather, 2012). In most cases, individuals remember only select content of negative experiences well. There remain debates about how best to characterize the mnemonic associations that are enhanced vs. impaired vs. unaffected by negative arousal. The distinctions may relate to how “intrinsic” the details are to the item (Kensinger, 2009; Mather, 2007) with features that are inherent to the stimulus, such as an object’s identity or color, prioritized in memory. For example, Palombo, Te, et al. (2021) designed an experiment in which participants viewed short video clips with inserted negative or neutral objects. Participants were asked to indicate whether they recognized the object, when during the video clip they had seen the object, and what other scenes had occurred within the same videoclip. Results revealed that negative emotion (compared to neutral) was related to improved recognition accuracy and temporal-order memory for the objects but poorer performance for choosing the scenes from the same videoclip. In other words, individuals remembered the emotional object from the film and approximately when in the clip it had appeared, but not the broader context in which the emotional object had appeared. The literature has more generally suggested that, for experiences with negative content, there may be a shift from prefrontally guided integration of content, which allows for retention of broader contextual information, and toward a reliance on sensory processing, which allows for retention of more item-specific detail (Bowen, Kark, & Kensinger, 2018). Content perceptually bound to the emotional item may be disproportionately remembered (Murray & Kensinger, 2014), while other aspects are forgotten.

The selective memory enhancements also may relate to the goal-states of the individual and the alignment of features with a participants’ encoding goals (Kaplan et al., 2012; Levine & Edelstein, 2009). For instance, when participants are explicitly instructed to process all elements of a scene (Kensinger & Schacter, 2008), they do better at remembering all elements of negative scenes, including the contextual details, compared with a naturalistic viewing condition; and when individuals are asked to unitize negative and neutral items together, creating a single, coherent representation, they are able to do so faster than for two neutral items (reviewed by Murray & Kensinger, 2013). In other words, when explicitly instructed to bind a contextual element to a negative item, individuals *can* use their encoding goals to do this more efficiently, but they do not appear to do so by default.

These memory enhancements for select aspects of experiences, and the corresponding evidence that contextual elements are often poorly remembered, is inconsistent with positive interactions between the amygdala and the hippocampus. Of course, the *modulation model* does not require that there are always these positive interactions, and indeed many have theorized of the amygdala and hippocampal memory systems as those that operate independently, except when they coordinate to support emotional memory (Phelps, 2004; Yang & Wang, 2017). However, there has been an alternate view in the human cognition literature for some time, with ideas of a “hot” emotional/fear, amygdala-driven system and a “cool” cognitive, hippocampal-driven memory system, with these systems being proposed to often act in opposition to one another (Metcalfe & Jacobs, 1996, 1998). Some studies of human memory are consistent with this idea of these systems acting in opposition, such as research demonstrating that while item memory is usually enhanced for negative relative to neutral stimuli, associative memory often is impaired (Bisby et al., 2016; Bisby & Burgess, 2014; Madan et al., 2012).

In their *emotional binding* model, Yonelinas and Ritchey (2015) proposed another alternative for the way to consider the roles of the amygdala and the hippocampus in emotional memory. They expanded upon an influential model of human episodic memory (Diana, Yonelinas, & Ranganath, 2007), in which the hippocampus serves to bind contextual details into an episodic memory representation, building in emotional memory by proposing that the amygdala separately acts to bind items to their emotional salience. Recent evidence to support this model has come from studies demonstrating that emotionality is only associated with an item when there is explicit recognition of that item (Bell et al., 2017; Palombo, Elizur, et al., 2021). In the absence of item recognition, individuals cannot remember whether snakes are poisonous or nonpoisonous (Bell et al., 2017), and a transfer of valence from a negative item to a neutral item seems to occur only if the neutral item was episodically bound to the negative item (Palombo, Elizur, et al., 2021). These results suggest the possibility that emotion may be inextricably bound to item representations in episodic memory and unable to be retrieved in the absence of those item representations.

A nice feature of the *emotional binding* model is that it does not require there to be opposition between the amygdala-binding and hippocampal-binding systems. It can allow for some situations in which amygdala-binding may take place at the expense of hippocampal-binding, which often is discussed when talking about the “weapon focus effect” (Loftus et al., 1987) and the tendency for negative memories to become separated from the context in which they occurred (Bisby & Burgess, 2017). The model also allows for other situations in which amygdala-binding may co-occur with hippocampal-binding, as may occur when emotional experiences are more likely to be remembered with their spatial (Schmidt et al., 2011) or temporal (Palombo, Te, et al., 2021) context, and as originally proposed by the *modulation model*.

### Strength of encoding contributes to memory’s durability

While the *modulation model* and the *emotional binding* model both focus on processes specific to emotional memories, Talmi (2013) compellingly reviewed evidence that emotional memories also benefit from mechanisms that are engaged for many experiences and simply enhanced for emotional ones. Talmi’s *mediation model* proposes that emotional experiences benefit from boosted attention, elaboration, and organizational processes implemented at encoding (Talmi, 2013). A key element of this model is that it can explain emotional memory enhancements that arise over short-term delays; it does not require time for consolidation processes to unfold.

There is evidence that, at least in younger adults (see Box 4 for discussion of older adults), the types of encoding processes outlined by the *mediation model* may be particularly likely to be engaged for negative information (Kang et al., 2014; Kensinger, 2008; Ochsner, 2000), perhaps in part because of the modes of cognitive processing that negative emotions catalyze (Schwarz & Clore, 1996; Storbeck, 2012; Storbeck & Clore, 2005). That is, we remember negative experiences well, because they are prioritized for processing, and we grant them more of the cognitive processes that are well-known to increase the likelihood that an event becomes a part of our memory representations. For instance, Talmi et al. (2008) provided evidence for the attentional mediation of the emotional enhancement of memory. They asked participants to view negative arousing or neutral pictures under conditions that varied the attentional resources granted to those images, and then they gave participants an immediate recognition memory test. Results revealed that the immediate emotional enhancement of memory was related to the recruitment of a region of the left fusiform gyrus that also was associated with increased attention to the negative images.

Evidence for the impact of encoding processes on negative memories’ durability has come from studies examining how emotion regulation impacts subsequent memory. Multiple studies have revealed that when participants are asked to engage in cognitive reappraisal—that is, to reframe an experience to make it less negative—memory for those experiences is enhanced (Kim & Hamann, 2012; Leventon et al., 2018). These studies can be thought of as testing the relative impact of the types of processes encompassed in the *mediation model* and those arousal-related processes emphasized in the *modulation* and *emotional binding* models. That is, cognitive reappraisal asks participants to grant additional attention and elaboration to negative stimuli (increasing those processes emphasized in the *mediation model*) to reduce the arousal associated with those stimuli (reducing the types of specialized mechanisms emphasized in the *modulation* and *emotional binding* models). The fact that this type of reappraisal leads to memory benefits for the negative content suggests the remarkable power of those cognitive processes that boost emotional memory via nonarousal-modulated mechanisms. They can enhance memory—including over a longer-term delay of two weeks (Kim & Hamann, 2012)—even while diminishing (though not entirely removing) the possibility of arousal-modulation processes.

Although the *mediation model* was proposed to explain the emotional memory enhancements over shorter time-frames, it is worth considering that these factors also could explain some aspects of the time-dependent enhancements for emotional information. It is plausible that when experiences (neutral or emotional) garner more attention, elaboration, and organization, this leads to increased rehearsal or reactivation over subsequent delays. Christianson (1992) emphasized the importance of rehearsal for the maintenance of emotional memories. It is well known that sleep, and other rest-filled delays, can serve as a time in which experiences are reactivated in memory, with some of the signals as to which memories ought to be reactivated coming from prioritization signals set up at encoding (reviewed by Payne & Kensinger, 2018). While these prioritization signals are often discussed in the context of emotional tagging, arising via arousal responses and amygdala activation (Richter-Levin & Akirav, 2003), prioritization signals can also arise due to goal-states related to anticipated future-relevance of stimuli. For instance, when students highlight material (Lo et al., 2016) or study material they know they will later be tested on (Bennion et al., 2016), those aspects are preferentially consolidated over sleep-filled delays. In at least some designs, these prioritization signals can even win out over any affective-tagging elicited by emotional content. For instance, Bennion et al. (2016) revealed that intentional encoding was such a strong boost to retention of information over a sleep-filled delay that, while emotional content was preferentially retained over sleep when it was incidentally encoded, the neutral items rose to nearly the level of the emotional items when they had been intentionally encoded for a memory test that participants knew would occur after the period of sleep.

### Summary of factors leading to negative memories’ durability

In summary, extensive research has shown that negative memories are more durable than other types of memories. Their durability likely stems from multiple factors: Negative experiences are granted additional cognitive resources during encoding, aiding memory in the short-term (*mediation model*; Talmi, 2013) and providing prioritization signals that increase the likelihood that those experiences are later rehearsed (Christianson, 1992) or reactivated (Payne & Kensinger, 2018) in ways that benefit their maintenance over the longer-term. Negative experiences can also trigger mnemonic mechanisms that more directly influence consolidation. Sometimes, increased engagement of the hippocampus may contribute to the memory’s durability over time (*modulation model;* McGaugh, 2000, 2004). Other times, amygdala engagement can be sufficient to trigger item storage in the absence of hippocampal engagement (*emotional binding model*; Yonelinas & Ritchey, 2015). Importantly, what is durably retained are *select* portions of the emotional experience: those item aspects that were bound to the emotional salience (*emotional binding model*) and those aspects that, through emotional salience or goal-relevance, won out in prioritized competition for encoding resources (*ABC and GANE models;* Mather & Sutherland, 2011, Mather et al., 2016; see Figure 2 for depiction of these models of emotional memory)*.*

## Negative Content is Prioritized at Retrieval and Vividly Recollected

We all have examples of exceptionally vivid memories for particularly emotional, significant, and distinct events in our lives. Highly arousing personal memories stand out among more neutral autobiographical memories because of this increased vividness (Berntsen, 2001; Bohanek et al., 2005; Reisberg et al., 1988) and accessibility over time (Waters & Leeper, 1936). Our intuition tells us that these memories should also be more accurate, but this is often not the case. A landmark study by Neisser and Harsch (1992) revealed that in cases of “flashbulb memories” (Brown & Kulik, 1977), individuals remain highly confident in their memory for the details of the event, even though those details can become degraded and distorted over time. Talarico and Rubin (2003) expanded upon this important finding, declaring “Confidence, not consistency, characterizes flashbulb memories.” In other words, during retrieval, individuals experience inflated confidence for emotional memories, believing their memory to be accurate even when objective metrics suggest otherwise.

Initially, these results seemed contradictory with evidence that negative memories were often associated with a greater sense of recollection: Both when the retrieval cues themselves are emotional (Kensinger & Corkin, 2003; Ochsner, 2000) and when memory is assessed for neutral items encoded in negative versus neutral contexts (Jaeger et al., 2009; Maratos & Rugg, 2001; Smith et al., 2004), individuals are more likely to report that they have a vivid, specific recollection of a past negative event than of a past neutral event. Indeed, there are dissociations between the subjective vividness of a memory and its accuracy (Brewin & Langley, 2019). Phelps and Sharot (2008) argued that one reason for this disconnect is because of differences in the way that details of emotional versus neutral memories aggregate to affect the subjective experience of recollection associated with those memories. For emotional memories, individuals may base their recollective experiences on the strength or quality of a few select details, while for neutral memories, individuals may base their recollections on a broader and aggregated set of details. Indeed, when thinking back on the most negative events from our personal past, we tend to focus on those aspects that we think of as the most central to the event, rather than peripheral details (Berntsen, 2002; Talarico et al., 2009). It is almost as if people do not realize that there are missing details (Phelps & Sharot, 2008; Sharot et al., 2004) and that the visuo-perceptual vividness of their negative memories is fading with time (Cooper et al., 2019). This focus on selective portions may arise because those aspects are associated with prioritized search and elaboration processes, but with reduced monitoring at retrieval (Figure 1). Individuals may end their search for event details prematurely, once the negative elements come to mind, or they may skip to elaborating on the negative elements without monitoring for the accuracy or completeness of the retrieved content. In other words, metamemory or memory monitoring failures may account for some of the overconfidence in these memories’ accuracy (see discussion in Krug, 2007).

Considering the *emotional binding* model, another way to understand this pattern may be that, at retrieval, there is a prioritization of accessing the details retained via amygdala binding, with less emphasis given to retrieving details retained via hippocampal binding. Although this remains speculative, it would be consistent with a mystery in the literature: There are robust trade-offs in memory when tested via recognition. That is, individuals do better at recognizing emotional elements within scenes than neutral elements, but they do worse at recognizing the contexts in which emotional versus neutral elements were presented (Kensinger & Corkin, 2004; Kensinger et al., 2007). However, if the task is switched from a recognition task to a cued-recall task, where individuals are asked to generate the context that had been paired with an object, or vice-versa, there is a cued-recall advantage for the negative scenes (Madan et al., 2020; Mickley Steinmetz et al., 2016). While future work is needed to elucidate the basis for this dissociation, one possibility is that the cued-recall instructions, by putting emphasis on the retrieval of the association, force a focus onto details stored via hippocampal binding; by contrast, recognition instructions, by enabling a reliance on item processing alone, may keep the reliance on those details retained via amygdalar binding. More generally, these results are important in showcasing that the degree of selectivity for negative versus neutral memories can sometimes be related to how details are brought to mind at the moment of retrieval rather than to whether those details exist at all within the memory trace.

These behavioral data—showing dissociations between recognition and cued recall, and demonstrating disconnects between metrics of subjective vividness and objective memory content—imply an effect of emotion on retrieval and retrieval monitoring. As described earlier, much of the initial focus into understanding the cognitive neuroscience of emotional memory was on the processes that unfolded as an event was experienced and initially consolidated. Within the last few years, there has been a more direct focus on the role of retrieval processes in giving negative emotional memories their power. Here, we describe two models that focus specifically on how negative memories are prioritized at retrieval (eCMR; Talmi et al., 2019) and recollected with sensory detail (NEVER Forget; Bowen, Kark, & Kensinger, 2018).

### eCMR: Negative Memories Crowd Out Neutral Memories

It is increasingly appreciated that associations are continuously being made between content and context—between information being learned or retrieved and the context in which those memory processes are unfolding (Lohnas et al., 2015; Polyn et al., 2009). It also has been demonstrated that the internal context of an individual is continually shifting, in ways that create temporal context shifts over time (Manns et al., 2007) and enable events to be linked via their shared temporal overlap (Cai et al., 2016).

A recent model, the emotional Context Maintenance and Retrieval Model (eCMR) (Talmi et al., 2019), has added emotion as a contextual dimension that can guide encoding and retrieval processes. This model accounts for the time-dependency of the emotional enhancement effect by proposing that emotional context can be more easily reinstated after delays than temporal or other contextual contexts. As predicted by context models of retrieval, this shared emotional context can lead to clustering effects in recall, whereby individuals will cluster their recall of items with negative emotional content even when those items were originally studied interspersed with neutral items (Barnacle et al., 2016; Long et al., 2015). However, the clustering effects have not yet been shown to relate to the degree of the emotional enhancement of memory, a pattern that would be expected were context effects at retrieval the primary driver of the memory enhancement.

An important revelation in studies comparing recall of items presented in pure-lists versus mixed-lists is that recall of emotional content often “crowds out” neutral content. That is, the reason why emotional items are remembered better than neutral items when studied in mixed lists, but not in pure lists, is largely explained by the fact that recall of neutral items is worse when appearing in mixed lists relative to appearing in pure lists. Although emotional items are sometimes better remembered in mixed lists compared to pure lists, this is not always the case (reviewed in Talmi et al., 2019). This crowding-out effect suggests that some of the improved accessibility of negative memories at retrieval may come from the fact that those memories are being selected at the expense of other neutral content and, once in mind, are setting up a context that will further bias the retrieval of additional negative content.

To our knowledge, eCMR has not yet been applied to paradigms that examine memory selectivity (e.g., memory for negative components at the expense of memory for neutral components of a photograph), but it seems plausible that the emotional context maintained could also be part of the explanation for the selectivity of memory. This ability for negative memories to crowd out neutral memories is reminiscent of trade-off effects, which have recently been attributed to retrieval effects as well as to encoding effects (Madan et al., 2020; Mickley Steinmetz et al., 2016). Future work will do well to examine whether retrieval context keeps memories honed onto the negative components while crowding out memory for other contextual details.

### NEVER Forget: Negative memories yield sensory specificity and vividness via recapitulation

It has long been known that memory is best when a person’s state at retrieval—be it internal or external—matches their state at encoding (Tulving, 1974), with experimental evidence dating back to at least the 1940s (Abernethy, 1940). eCMR launches from this premise, expanding from the idea that emotional context is present at encoding and is sufficiently long-lasting to be recapitulated at retrieval. Another model also launches from this premise: that recapitulation is central to episodic memory retrieval, and that the power of negative memories can be understood by considering what happens when negative events are recapitulated in memory.

Bowen, Kark, and Kensinger (2018) proposed that Negative Emotional Valence Enhances Recapitulation (“NEVER Forget”). When memories are negative, there is an increased likelihood that the brain reconfigures itself at retrieval to be in a similar state to the one it was in during encoding. The model was based on evidence that negative memories are associated with greater encoding-to-retrieval overlap in a number of regions, including in sensory-processing regions (Bowen & Kensinger, 2017a; Kark & Kensinger, 2015). In fact, even when using exclusively neutral prompts to cue memory for a previously encountered positive, negative, or neutral event, one of the strongest predictors of retrieval-related activity in sensory regions was the valence of the encoded event (Bowen & Kensinger, 2017b*).*

Key tenants of that model were tested by Kark and Kensinger (2019a, 2019b), who replicated the finding that sensory recapitulation was greater for negative than neutral or positive memories, and who further showed that the way sensory regions were incorporated into memory networks led to those differences at retrieval. In particular, as physiological responding increased during encoding, early visual cortex regions became functionally connected to the amygdala in a way that enhanced memory for negative, but not positive or neutral events (Kark & Kensinger, 2019b). In other words, increased arousal led to the incorporation of sensory regions into emotional memory networks specifically for negative stimuli. Moreover, when those sensory regions stayed incorporated into emotional memory networks post-encoding, as measured via resting-state connectivity, that led to a more sensory-driven retrieval of negative memories and to a greater propensity for participants to show a negative memory bias. Thus, some of what gives negative memories their power is their sensory specificity and their vividness via recapitulation.

Clewett and Murty (2019) have proposed that the neurobiological underpinnings of this selective memory phenomenon may come from the activation of an arousal-related locus coeruleus-norepinephrine system. They suggest that when sympathetic arousal and activation of this system is high, there is a prioritization of item features at encoding and a reinstatement of the corresponding lower-level sensory cortical regions during retrieval. By their model, it is not the negative valence of the experiences *per se*, but the arousal and behavioral activation they elicit, and their ability to engage the norepinephrine system, that drives their effects on recapitulation. How best to characterize these differences—whether they are primarily related to the aversive or pleasant nature of the experiences or to differences in the motivational states they elicit—remains an important point for further research.

### Prioritization of Negative Memory Retrieval

While the timecourse for these retrieval effects is still being investigated, extensive work has suggested that stimuli with high intrinsic motivational salience—often, high-arousal negative or threat-related stimuli—are prioritized for rapid access to retrieval processes. For instance, Jaeger et al. (2009) presented participants with items encoded in a scene with negative arousing content or with neutral content and later tested participants’ memories for the items while measuring event related potentials (ERPs); the key question was how the neural markers of retrieval would differ based on the previously studied encoding context of the items. Although the precise effects differed based on whether recognition was tested after 10 min or 24 hours, at both delay intervals, differences when recognizing items studied in a negative-arousing context versus a neutral context emerged around 200 ms, relatively early in the retrieval process and before markers of conscious recollection (see also Bowen, Fields, & Kensinger, 2018 for evidence of differences that emerged around 200 ms after retrieval-cue onset). Similarly, Righi et al. (2012) presented participants with images of faces with happy, fearful, or neutral facial expressions. Then, at retrieval, all individuals were presented with faces displaying a neutral expression, and ERP was used to measure the timecourse of responses as individuals indicated whether they recognized the face. Because all expressions were neutral at retrieval, any differences in timecourse would be based on the different facial expressions seen at encoding. Faces that had been studied with a fearful expression elicited ERP markers of enhanced visuo-attentional processing (a greater P100) and evidence of primed facial feature processing (a reduced N170 combined with a larger early frontocentral effect).

Negative memories may also enhance later recollective signatures and may engage later, postretrieval processes differently. For instance, Ventura-Bort et al. (2020) demonstrated that the late parietal old/new effect (occurring approximately 600-800 ms poststimulus) was evoked for old items previously associated with a (negative) emotional background. They additionally found that a waveform later in the retrieval epoch (800 ms and beyond) was enhanced for objects that had been encoded in that emotional context. Other ERP studies also have demonstrated that when images are unpleasant (Lavoie & O’Connor, 2013) or when neutral images are studied in negative contexts (Liu et al., 2021), those retrieval cues can modulate neural signatures later in the retrieval epoch. These types of results have led to the postulation that negative memories are associated with different retrieval orientations (Liu et al., 2021), with more sustained processing (Ventura-Bort et al., 2020), and with different levels of strategic control during retrieval (Herron, 2017). Together, these results suggest that there may be a privileged access to negative memories, and that once they are brought to mind, there is additional processing granted to the negative content of those memories.

## When the Power of Negative Memories is Maladaptive

The power of negative memories can differ across people and across situations, and under some circumstances can become maladaptive. We briefly review how the power of negative memories is altered in individuals with affective disorders, such as depression and posttraumatic stress disorder (PTSD). We focus specifically on the negative memory biases that often exist in these disorders and can correlate with symptom severity, and on the role that rumination also may play.

### Negative memory biases

Sometimes, the durability of negative memories can be problematic for mental wellbeing. In disorders, such as depression or PTSD, the way negative information is attended and interpreted is thought to play an important role (Ledoux & Muller, 1997), leading to negative memory biases that can contribute to symptom persistence (Harmer et al., 2009; Imbriano et al., 2022). Negative memory biases describe the *relative quantity* of events brought to mind, such that patients with depressive or PTSD symptoms are more likely to remember negative over positive or neutral experiences (Gibbs et al., 2013; Harmer et al., 2009; Imbriano et al., 2022), and this negative memory bias is thought to catalyze the onset of negative thinking and depressed mood (Harmer et al., 2009). For example, Imbriano et al. (2022) found that severity of PTSD symptoms, including depression, dysphoria, and panic attacks, was related to the tendency to remember studied negative material more accurately than studied neutral material.

Importantly, negative memory biases can result not only from enhancements in memory for negative events but also from reductions in memory for positive events. For instance, individuals with depression show relative impairments in memory for positive events compared with negative or neutral events, possibly because of dysfunctions in the dopaminergic system that would typically strengthen the encoding of these events into memory (Dillon, 2015; Dillon & Pizzagalli, 2018). Consistent with a dopaminergic hypothesis, acute administration of dopamine agonists has been shown to increase striatal activation in response to reward in individuals with major depressive disorder (Admon et al., 2017), and while there were not behavioral effects of that acute administration, longer-term administration of a dopamine agonist has shown beneficial for reducing symptoms of dysthymia or depression (Zangani et al., 2021). Similarly, in PTSD, pharmacological enhancement of cortical dopamine has shown some benefits for those with severe PTSD, reversing response biases toward fearful stimuli presented on a working memory task (Westphal et al., 2021).

### Rumination

Other times, it is not the durability of negative relative to positive memories that is problematic, but the inability to stop reflecting on particular past negative experiences: rumination. Rumination is present across many affective disorders, and while it may sometimes be deployed in the hopes of achieving an adaptive outcome (Lyubomirsky & Nolen-Hoeksema, 1993), it instead tends to exacerbate negative moods and encourage maladaptive problem solving (Nolen-Hoeksema et al., 2008; Watkins, 2009).

While rumination may sometimes be deployed strategically, it also may arise because individuals fail to effectively gate their memory retrieval processes. Fawcett et al. (2015) revealed that those who ruminate are more likely to have difficulties controlling the contents of their memory; that is, they struggle to put unwanted or unneeded memories out of mind. A recent meta-analysis (Stramaccia et al., 2021) supported the conjecture that this type of memory suppression occurred more robustly in healthy individuals than in anxious or depressed individuals. These results may explain why rumination can be both a risk factor for the development of PTSD and also correlated with the maintenance of PTSD symptoms (Ehring, Ehlers, & Glucksman, 2008; Ehring, Frank, & Ehlers, 2008). It is plausible that the intentional focus on a past negative experience, when combined with a difficulty later suppressing the repeated retrieval of that experience, is a recipe for an intrusive memory (Ball & Brewin, 2012).

## Modifying the Negativity of Memories while Maintaining their Power

Sometimes, a person can diminish the negativity of a memory, while retaining some of its power. We describe how the power of negative memories can be modified via engagement of emotion regulation strategies that change how individuals feel in response to an event (i.e., during event encoding: Gross, 1998) or a memory (i.e., during event retrieval: Holland & Kensinger, 2013a). We focus primarily on studies with nonclinical samples, but include brief discussion of connections to some commonly used therapies.

### Emotion regulation at encoding

It is well known that the intensity of a negative event can be manipulated at encoding, by engaging in emotion regulation processes. A variety of strategies can be implemented, with different efficacy in-the-moment and with different consequences for later memory (Gross, 2002). Of the regulation strategies, cognitive reappraisal is usually discussed as being one of the most helpful. It works in-the-moment and is associated with better metrics of mental wellbeing than many other strategies, such as suppression or avoidance. As we described earlier, in the context of our discussion of the *mediation model*, an interesting aspect of cognitive reappraisal at encoding is that it can help to preserve memory for the *content* of an experience while stripping some of its affective intensity (Dillon et al., 2007; Richards & Gross, 2000). This pattern extends to autobiographical experiences: college students’ use of reappraisal just after a negative event corresponded with better memory performance and with a tendency to later underestimate the emotional impact of the event (Colombo et al., 2021). When you want to remember the critique given to you by a coworker, while not being overcome with negative emotion during the interaction or when reflecting on it later, cognitive reappraisal may be the strategy of choice.

The neurobiology that makes this possible is still not fully understood. It is clear that cognitive reappraisal engages prefrontal processes, often in the service of downregulating amygdala activity (Banks et al., 2007; Goldin et al., 2008). It may be that, by engaging those prefrontal processes that also serve to deepen the level-of-processing associated with encoding, memory is enhanced (Pannu Hayes et al., 2010). It also is plausible that part of the benefit to memory comes from reducing the experienced negative affect: If negative emotions shift the balance from a hippocampal-binding system toward an amygdala-binding system (see Box 3), then using reappraisal to weaken those negative emotions could help to keep the hippocampal-binding system engaged, increasing the likelihood that memories are able to be richly recalled and also decreasing the likelihood that they are recalled in maladaptive ways divorced from the encoded context. Thus, emotion regulation at encoding has the interesting potential to reduce the power of negative emotional memories insofar as it will reduce the intensity of the experienced negative emotion at the time of the experience and reduce the likelihood of maladaptive retrieval.

**Table:** 

**Box 3. Amygdalar and Hippocampal Binding Systems** The modulation model proposes that arousal, and specifically norepinephrine release, triggers **cooperation** between the amygdala and the hippocampus (Roozendaal & McGaugh,^ 2011^). Other research suggests that negative emotion can trigger **disconnects** between amygdala engagement and hippocampal engagement, consistent with behavioral evidence that negative emotion can lead to memories that are less coherent**,** with fewer within-event associations (Bisby & Burgess, 2017; Madan et al., 2017; Palombo, Elizur, et al., 2021). For instance, when individuals studied face-occupation pairs, the presence of negative occupations were associated with lower hippocampal engagement during encoding and with poorer memory for those associations (Berkers et al., 2016). Bisby et al. (2016) similarly found that the encoding of negative items was associated with a boost in amygdala activity but with a decrease in hippocampal activity, corresponding with an increase in item memory but a decrease in associative memory for those negative items. Yonelinas and Ritchey’s (2015) *Emotional Binding* model may provide a framework in which to understand these seemingly conflicting results. By this model, there are two binding systems at work during encoding: an amygdala-based system, that prioritizes binding the item to its emotion, and a hippocampal-based system, that prioritizes binding the item to its context. It is plausible that there are situations in which **both systems** are engaged, leading emotional items to be remembered in their broader context, and situations in which it is **primarily the amygdala system** that is engaged, leading emotional items to be remembered void of their context. An intriguing possibility to be addressed by future research is that negative emotion may create an imbalance between engagement of the amygdala and hippocampal systems and a shift toward amygdala binding, while positive emotion may be more likely to lead to simultaneous engagement of both systems or even a shift toward hippocampal binding.	

### Emotion regulation at retrieval

Not only can *experiences* be regulated, but also *memories* of those experiences can be regulated. This may be critically important to our mental health, If in the moment we fail to effectively regulate our responses to an event, later, as we reflect on the event, we can have another opportunity to reframe the experience (see review by Samide & Ritchey, 2021).

Individuals can intentionally and strategically regulate their responses to their memories, such as when individuals are specifically instructed to reduce their emotional reactions to a memory of an encoded negative image or a negative autobiographical memory (Holland & Kensinger, 2013a, 2013b). In these instances, it seems that individuals bring the past content into mind and then, similar to emotion reappraisal at encoding (Morawetz et al., 2017; Ochsner & Gross, 2005), engage lateral prefrontal control processes to down-regulate emotion regions and perhaps also sensory regions. There can be lasting consequences to this type of reappraisal, with individuals continuing to rate the memories as less emotionally intense even after some time has passed (Holland & Kensinger, 2013a, 2013b).

Strategic cognitive reappraisal may be similar to those processes encouraged in various forms of therapy (Kredlow et al., 2018). Broadly, cognitive behavioral therapy teaches techniques that patients can use to reframe negative thoughts as more positive ones (Coffey et al., 2015). Cognitive restructuring techniques more specifically encourage retrieval of a negative past experience, with the goal of the clinician guiding the rememberer toward a reinterpretation of the event’s meaning (Beck, 2011). Imagery rescripting similarly involves the retrieval of a past negative event coupled with the retelling of the memory with the inclusion of more positive and less negative imagery; this method has shown promise for multiple affective disorders linked to maladaptive negative memory retrieval (Morina et al., 2017).

While we have so far described methods that direct individuals to reframe or regulate their memories, aversive memories also may be spontaneous regulated. It is unclear if most people are able to engage in spontaneous regulation or if it primarily occurs in those individuals who are chronically motivated to reduce their experience of negative affect. In contrast to lateral prefrontal regions that may serve an outsized role in the strategic reappraisal of memories, the dorsomedial prefrontal cortex (dmPFC) may be particularly important for this type of spontaneous regulation. Kensinger and Ford (2021) have recently proposed that, at each phase of memory, the dmPFC participates in integrating the affective components of an experience with its other content. Through its various connections, including with the hippocampus (Ford & Kensinger, 2018), it may be able to play a key role in orchestrating memory framings that will either emphasize or deemphasize affective components. At retrieval, this may enable the dmPFC to down- or up-regulate the vividness of memories for negative images (Ford & Kensinger, 2017) or to dampen or intensify the focus on negative details of mixed-valence autobiographical events (Ford & Kensinger, 2019), even in the absence of explicit emotion-regulation instructions.

Finally, memories can be regulated by biological means. Rimmele et al. (2015) had participants read negative and neutral texts and then, 3 days later, recall the texts with either pharmacological suppression of cortisol levels or naturalistic levels. They found that when cortisol was suppressed, retrieval of the negative texts was impaired, with no corresponding impairment for the neutral texts. Importantly, these changes were long-lasting, with poorer memory for those negative texts persisting 1 week later. Although in this study, the regulation of cortisol happened via pharmacological intervention, it also is plausible that individuals could regulate their cortisol levels via naturalistic means, thereby weakening the strength of negative memories retrieved in that altered state.

Importantly, both the pharmacological work and the broader work on emotion regulation during retrieval (Holland & Kensinger, 2013a) suggests that the effects extend beyond the single occurrence during which the negative memory retrieval is regulated. Possibly by affecting the way that memories are reconsolidated after initial retrieval (Drexler & Wolf, 2017), once a memory is altered via regulation at retrieval (strategic, spontaneous, or biological), there can be lasting consequences for its content. We will return to a discussion of how negative memories may become more positive over retrievals when we discuss the power of positive memory retrieval.

Although future work is needed, it is possible that the timing of regulatory processes during retrieval is important. For instance, Holland and Kensinger (2013a, 2013b) found that when individuals were instructed to *increase* their emotional reactions to a negative memory, it was activity at the time those instructions were received—and before the memory prompt had appeared—that best corresponded with their success in upregulating their emotional reactions. Perhaps relatedly, a recent study (Bridgland & Takarangi, 2021) found that warning participants that retrieving a negative memory would be upsetting (designed to mirror “trigger warnings”) led those individuals to report a *greater* negative impact of the event than individuals who did not receive that warning. Thus, it is possible that part of what makes negative memories powerful relates to whether, before we have brought the memory to mind, we anticipate the impact of that recollective experience.

## The Power of Negative Memories for the Future

Kensinger and Ford (2020) noted that although emotional memory retrieval often is measured as an end-point, as the culmination of processes that allow content to be accessed, it also is a starting-point. When we retrieve a memory, we create an opportunity to modify the memory representation: we may embellish some details and diminish emphasis on others, and we may reframe an experience, perhaps incorporating new information that changes our earlier interpretation. These effects can be long-lasting: Retrieval is a starting-point in the cycle of a memory, with the way a memory is retrieved at one time-point influencing how it is re-encoded, and how related content is encoded and how the memory is updated. The memories that come to mind can impact small decisions (Do we return to a restaurant?) and larger ones (Do we accept the invitation to present at a conference? Do we go on a second date?). The details that we recall also can affect our mental wellbeing and can influence our ability to effectively empathize with others.

**Fig. 3:**
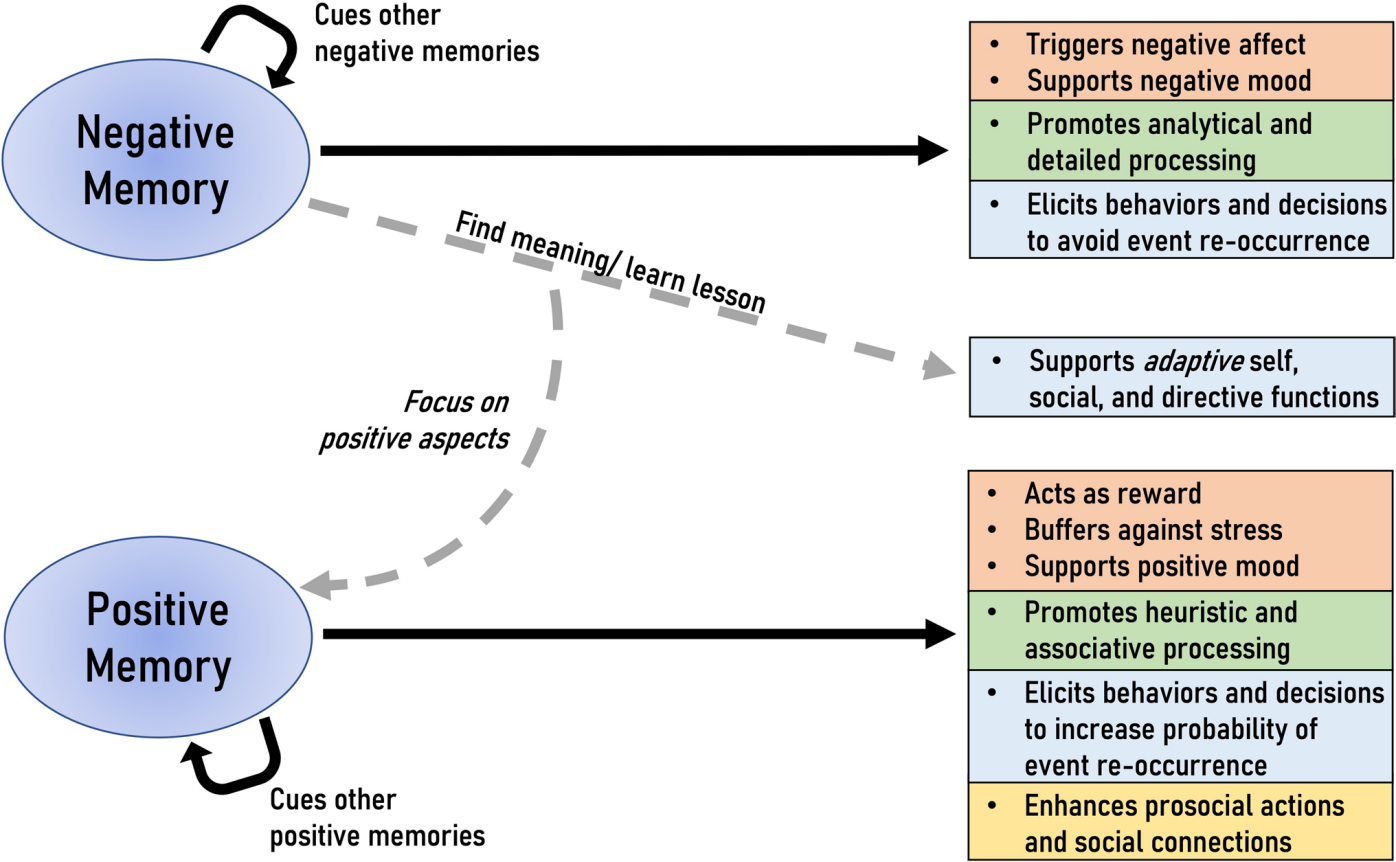
Consequences of Negative and Positive Episodic Memory Retrieval. The way a past negative or positive event is brought to mind has consequences across multiple domains. These memories can influence a person’s current affective state (denoted by peach color) and the way incoming information is processed (in green). They also can be used in directive ways, to guide actions and decisions (in blue) and, particularly in the case of positive memories, their retrieval can lead to prosocial behaviors (in yellow)

Across many of these domains, negative content holds particular power. The Availability Heuristic describes the tendency for decision-making to rely on only a small subset of the total relevant information (Tversky & Kahneman, 1974); while there are many features that influence what content is used, content having negative valence is high among them, leading people to overestimate the likelihood of events, such as a terrorist attack (Sunstein & Zeckhauser, 2011). More generally, our negative autobiographical memories can serve important directive functions: informing, guiding, and motivating our current actions (Rasmussen & Berntsen, 2009). This directive function is often discussed as being *adaptive* (i.e., helping us to better navigate the future), which often occurs by being able to extract some lesson from a past negative experience (Figure 3). For example, memory for a particularly negative event (i.e., “rock bottom”) could serve as a turning point, leading us down a more successful path (i.e., an *adaptive* function; Pillemer, 2001, 2003; Habermas & Bluck, 2000). Even the most traumatic experiences can sometimes serve adaptive self, social, and directive functions (Pillemer, 2003; Rasmussen & Berntsen, 2009), a phenomenon known as *posttraumatic growth* (Schuettler & Boals, 2011; Tedeschi & Calhoun, 2004). However, recent research suggests that memories for negative events also can serve maladaptive functions (Burnell et al., 2020); for instance, that memory of “rock bottom” could cause us to give up on our more challenging goals (i.e., a *maladaptive* function). Thus, it is important to consider that negative memories, depending on how they are interpreted at retrieval, have the potential to serve either adaptive or maladaptive functions that will alter how decisions are made.

Interestingly, one domain in which the negative does not seem to win out is in the domain of future prospection. When individuals think about the future, it often is positive events that are envisioned (D’Argembeau & Mathy, 2011; Rasmussen & Berntsen, 2013). People are slower to come up with negative future events than positive ones (Newby-Clark & Ross, 2003) and, while highly negative events are recalled from past time periods, the future projections more likely to be remembered are those that are positive (Gallo et al., 2011). Given that there is an immediate causal effect of positive future prospections on mental wellbeing (Grant & Wilson, 2021), it may be adaptive for individuals to envision a rosier view of their future (MacLeod & Conway, 2005). Indeed, despite so much research focused on how individuals remember negative experiences, memory for the good events from our past can hold tremendous power.

## What Gives Positive Memories Their Power?

Positive memories gain power from many of the same factors that give negative memories their power: They are long-lasting and highly accessible. While positive memories may not show the same automaticity of retrieval mechanisms as negative events, positive events from our personal past come to mind more frequently than negative events and can do so involuntarily (Walker et al., 2003). They also are richly associative, which may increase the likelihood that retrieval of one positive memory cues another.

Positive memories also hold a power all their own. Unlike the affect associated with negative memories, which tends to fade relatively quickly, positive memories are more likely to retain their affective intensity (Walker et al., 1997; see recent review by Skowronski et al., 2014). This may be part of the reason why positive autobiographical memories act as rewards in themselves (Speer et al., 2014) and can buffer effects of stress (Speer & Delgado, 2017). Memories for positive personal events become more integrally tied to our sense of self and can perpetuate self-esteem (Çili & Stopa, 2015) and become an important part of our life story (Berntsen et al., 2011).

Given these features of positive autobiographical memories, it may come as no surprise that they have high utility and can be strategically recalled to good purposes (Figure 3). Positive memories are powerful in their ability to repair our moods after a negative mood induction (Joormann et al., 2007; Joormann & Siemer, 2004), to connect us socially (Rasmussen & Berntsen, 2009; Wolf & Demiray, 2019), and to inspire us toward prosocial behavior (Gaesser & Schacter, 2014). By activating reward circuitry, they even may trigger mnemonic circuitry that increases the likelihood that we encode the good in the world around us. We will review the literature shedding light on the power of positive memories.

## Positive Memories are Durable

When we described the power of negative memories, we described the shallower forgetting curve for those events compared with neutral events. Studies of autobiographical memory demonstrate that positive memories also can show a shallower forgetting curve. We do not just remember a hair in our food or a classmate tripping over our backpack; we also remember a dessert accompanied by a birthday candle, or a classmate returning our dropped earbuds. Indeed, individuals can form flashbulb memories for positive events (Scott & Ponsoda, 1996), and many of the qualities of flashbulb memories can extend to personal events with high positive valence, such as when college students recall being asked to join a sorority or fraternity (Kraha & Boals, 2014). Positive memories also can be harder to put out of mind; in one recent study, individuals found it harder to direct themselves to forget positive social feedback relative to negative feedback (Xie et al., 2021).

## Positive Memories are Associative

The characteristics of memories for highly positive versus highly negative experiences are not always identical. Most notably, several reports have suggested that individuals who feel positively about the outcome of an event recall its details confidently but with less factual accuracy (Bohn & Berntsen, 2007) or with less consistency over time (Kensinger & Schacter, 2006b; Holland & Kensinger, 2012) than do individuals who feel negatively about the outcome (but see Chiew, 2021 for no effect of valence). In other words, while negative emotional memories can be subject to distortion (Pesta et al., 2001) and overconfidence (Talarico & Rubin, 2003), these effects can be exaggerated for positive memories. For example, Holland and Kensinger (2012) found that adults recalled details of the 2008 Presidential Election more consistently over time when they perceived the outcome as negative compared with positive. These results are consistent with laboratory studies that suggest that memory for positive experiences often is associated with generally knowing that an event occurred rather than being able to recall specific details (Kensinger, 2009; Kensinger & Kark, 2018; Ochsner, 2000) and that memory for negative experiences can be associated with more sensory specificity, whereas memory for positive experiences can include more of the conceptual framing or gist (Kensinger, 2009).

The experience of remembering a positive personal event can feel quite different than that of remembering a negative event. Positive autobiographical memories often are associated with increased ratings of vividness and of reexperiencing the original event during retrieval compared with negative events (Ford et al., 2012; Talarico et al., 2004). Furthermore, while memories of negative autobiographical experiences tend to exhibit strong item memory at the expense of associations, memories of positive experiences seem more likely to retain contextual associations (Talarico et al., 2009). For instance, Zimmerman and Kelley (2010) found that when participants were asked to recall which neutral, negative, or positive words had been paired together, cued recall was better for positive pairs than for neutral or negative pairs. Madan et al. (2019) replicated this finding and showed that this improved association memory was greater when two positive stimuli were paired together than when a positive word was paired with a neutral word.

Perhaps relatedly, positive emotion appears to consistently enhance prospective memory. Prospective memory, which is the ability to remember to complete a task or behavior in the future, requires associating an intention to perform some action with a cue that occurs later in time—typically an event-cue (when driving past the store, make a stop to pick up milk) or a temporal-cue (at 5 pm, take medicine) (Crystal & George Wilson, 2015; McDaniel & Einstein, 2007; Shum et al., 1999). A recent meta-analysis revealed a main effect of positive emotion on prospective memory performance, in that performance improved for positive cues compared to negative or neutral cues. The enhancement effect occurred when positive cues were used during both encoding and retrieval. Furthermore, positive emotion cues additionally enhanced prospective memory for older adults compared with younger adults (Hostler et al., 2018).

Although research is limited, modulation of the dopamine system has been purported as a mechanism supporting prospective memory (Costa et al., 2008a) and at least some forms of associative memory (Lee et al., 2021). For instance, in Parkinson’s disease patients, who show prospective memory deficits, receiving an acute dose of levodopa led to better performance on a time-based prospective memory task (Costa et al., 2008b). Increased connectivity between the ventral tegmental area (i.e., the origin of dopaminergic transmission within the mesocorticolimbic system) and hippocampus lead to enhanced associative memory (Tompary et al., 2015). Thus, release of dopamine may boost associative processing and contribute to some of the cognitive consequences of positive affect (Ashby et al., 1999), although debates remain about the connections between dopaminergic transmission and positive emotion (Goschke & Bolte, 2014).

These memory patterns are generally consistent with the broaden-and-build theory of positive emotion (Fredrickson, 1998), which posits that positive emotions during an experience allow an individual to holistically process an event and to use the influx of information to identify actions that can be taken and resources that can be used to respond. As a result, the recollection of that event is more general and heuristic. In line with the broaden-and-build theory of positive emotion, some studies suggest positive emotions allow individuals to think more flexibly and creatively (Ashby et al., 1999; Isen et al., 1987; Sacharin, 2009). In this context, the memory results—suggesting that relative to negative memories, positive memories may retain less specific detail about any particular feature but may include more associative connections—would be consistent with the idea that positive emotions help participants to process information more holistically and to draw creative connections.

We have previously proposed that these differences may arise from how sensory (for negative) versus prefrontal (for positive) regions are incorporated into emotional memory networks (Bowen, Kark & Kensinger, 2018). Relative to negative memories, the encoding and retrieval of positive information tends to be associated with increased activity in prefrontal regions, both medial and lateral, and in midline regions including the posterior cingulate and precuneus (Erk et al., 2003; Ford et al., 2014; Kensinger & Schacter, 2008; Mickley & Kensinger, 2008; Ritchey et al., 2011). Reliance on prefrontal structures for positive memory may also explain the benefits to prospective memory, which also is thought to rely on prefrontal engagement (Burgess et al., 2011; Volle et al., 2011).

Individuals who have stronger prefrontal-amygdala connectivity also may show a greater tendency to remember positive experiences. This association has been revealed in older adults, with greater medial prefrontal-to-amygdala connectivity corresponding with the degree of a positivity bias in memory (Sakaki et al., 2013). Among younger adults, there can be a relationship between amygdala-prefrontal connectivity and the tendency to remember positive events (Kark & Kensinger, 2019b). The ability to use neurofeedback to increase the strength of this prefrontal-amygdala connectivity during retrieval of positive memories can even be linked to remission of symptoms of depression (Young et al., 2018).

Although fMRI studies cannot speak to the necessity of these regions for positive memory, two studies using repetitive transcranial magnetic stimulation suggest there may be causal links between prefrontal engagement and memory for positive information. In particular, these studies provide additional support for the argument that retrieval of positive memories is associated with activity in prefrontal cortex, having found that stimulating dorsolateral prefrontal cortex activity during retrieval can improve accuracy and reduce response times for positive compared to negative memories, even in subjects with high levels of anxiety (Balconi & Ferrari, 2012, 2013). Improved positive memory performance from increased prefrontal engagement is broadly consistent with the more heuristic and conceptual memory representations that individuals seem to retain for these experiences. A link between positive memory and frontal function also may be suggested by the fact that positive emotion enhances prospective memory, and prospective memory is known to rely on anterior prefrontal engagement (Burgess et al., 2003; Reynolds et al., 2009).

Although it is speculative at this point, an intriguing possibility is that positive versus negative memories may be associated with differences in how amygdala-binding and hippocampal-binding systems coordinate. While negative memories may be associated with enhanced amygdala-binding mechanisms, they also can be associated with reduced hippocampal-binding (reviewed by Bisby et al., 2020; see Box 3). By contrast, the behavioral data may suggest that positive experiences do not create that same opposition. Perhaps, for positive memories, there is amygdala-binding *and also* hippocampal-binding. While the bound contextual details may be lacking in some resolution due to their processing at a more heuristic level, positive memory representations may be more likely to contain those hippocampal-bound contextual details as well as the amygdala-bound emotional salience. This proposal is in many ways in line with the dissociation proposed by Clewett and Murty (2019), who suggested that activation of the locus coeruleus-norepinephrine system leads to high memory selectivity while activation of the dopaminergic-ventral tegmental area (VTA) system leads to a more integrative memory representation. They centered this dissociation more on the allocation of attentional resources and the nature of sensory processing. But it is possible that, complementary to these effects, are effects on the balance of binding mechanisms engaged. It is plausible that VTA projections to both the hippocampus (Murty & Adcock, 2014) and amygdala (Tang et al., 2020) enable these binding mechanisms to act synergistically rather than in opposition (pushing memory representations toward the balanced-scales example in Box 3 rather than to an amygdala-biased representation). Future work will be needed to address this possibility.

Valence-related shifts in hippocampal- and amygdala-binding may help to explain the differential effects of arousal on positive and negative autobiographical memories. As discussed previously, positive autobiographical memories tend to contain more contextual information than negative memories (Berntsen, 2002; Talarico et al., 2009), leading to representations that are rated as richer and more vivid (Talarico et al., 2004). This effect of positive valence is independent of emotional arousal (Ford et al., 2012), suggesting that it may be supported, in part, by associative hippocampal processes. In contrast, the enhancing effect of negative valence on autobiographical memory has been shown to be dependent on arousal, only showing links to increased vividness and specificity for memories rated as highly emotional (Ford et al., 2012). In other words, the enhancing effects of autobiographical memory negativity may rely on more specific amygdala-binding systems that are triggered by increased arousal.

In addition to affecting the features of a successfully retrieved event, engagement of hippocampal- versus amygdala-binding systems may have implications for how memories are used for decision-making. While extensive research has framed decision-making as reliant on a running average compiled from past experiences, more recent work has highlighted that there are also many instances in which decision-making is more directly dependent on hippocampal-dependent episodic-memory mechanisms. For instance, Bornstein et al. (2017) revealed that incidentally reminding people of specific past decisions could bias their current decisions. This type of pattern suggests that decision-making is not relying on an average across past experiences but rather a sampling of past choices that can be influenced by the memories that are most accessible at a particular moment. Moreover, Murty et al. (2016) discovered that individuals could use past information to adaptively guide current decisions only when they had associative memory for the value associated with each item; item memory was insufficient. For example, it was only when a participant remembered which faces had been fair or unfair partners in a Dictator game that they were able to use that information to guide their decisions as to whom to select as a partner. More recently, FeldmanHall et al. (2021) connected this ability to adaptively choose partners to a trace signal in the hippocampus. Taken together, this burgeoning literature has led to new proposals for the role of the hippocampus in decision-making. Its role may predominate in situations in which a small number of past experiences are relevant, and also in cases where individuals must weigh different pros and cons, requiring an integration across multiple past experiences (He et al., 2022). In fact, some have gone so far as to suggest that the hippocampus’ ability to flexibly integrate across events gives it a central role in decision-making, even in circumstances that may not seem to have a dominant role for memory (Biderman et al., 2020), consistent with modern framings of the hippocampus as being specialized for *guiding future behaviors* more than for *remembering past experiences* (Biderman et al., 2020; Rubin et al., 2014). If true, then the engagement of the hippocampal-binding system could have important implications that transcend memory.

## Positive Memories Retain Their Affective Strength and Act as Rewards

Positive memories show not only a shallow forgetting curve *for the event* but also show a shallow forgetting curve for the *affect* of the event. While the affect of negative memories dissipates over time, the affect of positive memories tends to remain strong, a phenomenon referred to as the Fading Affect Bias (FAB). For example, in one study that examined the trajectory of memories’ affective intensity over a one-year period (Ritchie et al., 2009), it was found that *fading affect* was the most likely trajectory for negative memories, while *fixed affect* (i.e., unchanged over time) was the most likely trajectory for positive memories. Skowronski and colleagues pointed out that the FAB reflects not only this tendency for the affect of a positive experience to stay associated with the memory for a longer period of time than the affect of a negative experience but also the increased tendency for a negative event to eventually trigger a more positive emotion (Skowronski et al., 2014; Walker & Skowronski, 2009). Someone may be devastated at the time of a breakup, and only later come to realize that the relationship was not a healthy one. With time, we cannot only come to appreciate that things were not as bad as they initially seemed but also to appreciate the silver linings (Ford et al., 2016; Ford et al., 2021; see Box 4 for discussion of how older adults may be particularly good at this).

**Table:** 

**Box 4. Older adults’ memory for the positive** While the negative can often win out in younger adults’ memories, older adults are more likely to show a focus on the positive (reviewed by Mather & Carstensen, 2005; Carstensen & DeLiema, 2018). This effect is often referred to as the age-related “positivity effect,” and the pattern often is interpreted as arising from age-related changes in motivations, goals and preferences (Carstensen et al., 1999). The positivity effect in older adults’ memories has been observed in a variety of experimental paradigms and for a wide range of stimulus types. Older adults have better memory for positive information over negative information in tasks employing emotional images, word lists, and faces (Reed & Carstensen, 2012), and a meta-analysis revealed that the positivity effect is larger when cognitive processing is not constrained by the task instructions and when the age difference between younger and older adult groups is more extreme (Reed et al., 2014). It has more recently been demonstrated that not only do older adults remember proportionally more positive experiences than younger adults, they also can have an improved ability to focus on the positive aspects of otherwise-challenging life events. In a series of studies, Ford and colleagues demonstrated that, as compared to younger adults, older adults use more positive words to describe past events, even those that were viewed as quite negative at the time (Ford et al., 2016). After experiencing the 2013 Boston Marathon Bombing, older age was associated with an increased tendency to focus on the good that had come from the event (the heroism, the city coming together; Ford, DiBiase, & Kensinger, 2018), and 6 months later, older age also was associated with a decreased tendency to focus on the negative aspects of the event (Ford, DiBiase, Ryu, & Kensinger, 2018). A similar pattern was recently shown for reflections on the initial wave of the COVID-19 pandemic: older age was associated with an increased tendency to focus on the positive aspects (Ford et al., 2021).	

Because the affect associated with positive experiences does not fade quickly, this allows individuals who recall positive past experiences to relive those pleasant feelings. Retrieval of positive memories is regularly used in laboratory experiments to manipulate mood, and multiple studies have shown its effectiveness in doing so (Gillihan & Farah, 2005; Siedlecka & Denson, 2019). For example, after a negative mood induction, participants are able to use the retrieval of positive memories to boost their mood, an effect termed the mood-repair effect (Joormann et al., 2007; Joormann & Siemer, 2004). There also are broader relations between the retrieval of positive memories and the increased experience of positive affect (Joorman et al.,  2007) and life satisfaction (Hendriks et al., 2019).

More recently, it has been suggested that positive memories can literally be processed as rewards (Speer et al., 2014). When participants were asked to recall positive and neutral autobiographical memories, there was increased activity in corticostriatal circuitry typically associated with reward processing. Moreover, participants were willing to forego a small monetary reward in order to have the opportunity to recall a positive memory. The authors concluded that through their evocation of positive feelings and their engagement of reward-related regions, the recollection of positive experiences may be intrinsically valuable to an individual (Speer et al., 2014).

Speer and Delgado (2017) then went one step further, testing the hypothesis that positive memories can be used as a buffer for the effects of negative experiences by comparing the stress responses of individuals who recalled a positive or a neutral memory. Participants first underwent a Socially Evaluative Cold Pressor task (Schwabe et al., 2008). They then retrieved either positive or neutral autobiographical memories. Results were consistent with the buffering hypothesis of positive memories; individuals who retrieved positive memories had a smaller cortisol response to the stressor than did individuals who retrieved neutral memories, and they also reported less negative affect. Neuroimaging results, which showed increased prefrontal activity and connectivity among those who recalled positive memories after stress, led the authors to speculate that positive memories may serve emotion-regulation functions. In a follow-up study, Speer and Delgado (2020) showed that recalling positive memories with a social component could be particularly powerful in reducing the cortisol response following the same stressor task. These social-positive memories led to particular increases in activity in reward-related regions, emphasizing the impact of social engagement on positive memory recall and suggesting that positive memories that involve friends or family may provide additional resilience following stressful experiences.

Potentially related results have come from studies that looked at how nostalgia—a predominantly positive social emotion that arises from fond memories of one’s past (Sedikides et al., 2015)—can create analgesic effects. When people suffering from chronic pain wrote about an event that made them feel nostalgic (compared with an ordinary event that did not evoke such emotion), they reported lowered pain levels. Furthermore, college students who did not suffer from pain disorders were able to tolerate higher levels of applied pressure (i.e., showed higher pain tolerance) after writing about a nostalgic event (Kersten et al., 2020). This analgesic effect of nostalgia was recently confirmed in an fMRI study that showed participants images of objects or scenes designed to elicit nostalgic feelings of their childhood or to remind them of modern life (Zhang et al., 2022). During the viewing of the images that cued memories of childhood, participants reported more nostalgia, and when a painful stimulus followed those images, participants perceived it as less painful than when it followed the cues to modern life. FMRI results revealed that connectivity between the dorsolateral PFC and periaqueductal gray (a region linked to pain and analgesia; Linnman et al., 2012; Grahl et al., 2018) during the viewing of the nostalgic images related to this diminished perception of pain. Taken together, these studies suggest the fascinating possibility that retrieval of a positive memory can have retrograde and anterograde effects, minimizing the negative impacts of a just-experienced event (Speer & Delgado, 2017) or an about-to-be experienced event (Zhang et al.,  2022).

Retrieving a positive memory may not only itself serve as a reward but also influence how patiently people wait for a future reward (Lempert et al., 2017). When faced with a choice between smaller, immediate gains and larger long-term benefits (i.e., intertemporal choices; Strotz, 1956), individuals who were asked to retrieve positive autobiographical events (Lempert et al., 2017) or to imagine specific positive future events (Peters & Buchel, 2010; Benoit et al., 2011) were more patient, opting more often for long-term benefits (i.e., reduced temporal discounting). Similar shifts in temporal discounting are not seen when participants are asked to retrieve negative autobiographical memories (Lempert et al., 2017), imagine specific negative future events (Liu et al., 2013), or imagine novel positive scenes related to their positive memories (Lempert et al., 2017), suggesting that both positive affect and episodic construction are critical to these effects. There is further neuroimaging evidence to suggest that not all positive autobiographical memories influence temporal discounting to the same extent. During positive memory retrieval, activity in regions associated with reward processing, such as the striatum, has been linked to more patient choices (Lempert et al., 2017). This association suggests that positive autobiographical memories may have more power to influence subsequent behavior when retrieval is more rewarding.

## The Power of Positive Memories for Ourselves and for Our Future

We have already described the ability for positive memories to serve as rewards (Speer et al., 2014) and to serve important mood-enhancement functions (Bryant et al., 2005; Wolf & Demiray, 2019). Although these literatures have not been directly connected, it seems plausible that part of the power of positive memories for mood repair stems from their ability to act as an in-the-moment reward.

While retrieval of positive memories has the ability to transiently change how we feel, ongoing research is examining if the effects of positive memory recall are long-lasting. There is great interest in this topic across a range of subfields, with investigations of whether post-trauma mental-health indicators are tied to the accessibility of specific positive memories (Contractor et al., 2019) and whether positive-memory retrieval manipulations can benefit those with PTSD (Contractor et al., 2018, 2022). A recent systematic review of interventions conducted over the last twenty years (Miguel-Alvaro et al., 2021) described 12 intervention types, across 3 categories: techniques to increase access to and focus on positive memories; techniques to change qualities or features of positive memories; and techniques to improve self-esteem or emotion regulation. Specific methods employed included writing down positive autobiographical memories, describing the feelings and thoughts associated with a particular positive memory, and manipulating the vividness of recalled positive memories through narration to a therapist. Many interventions resulted in improved positive affect and reduced symptoms of depression. However, many of these effects appeared to be transient, and were not maintained at follow-up. The authors note that most assessments of these interventions lacked large sample sizes, replication studies, and longitudinal designs. Thus, future work is needed to examine the long-term utility of the use of positive memories.

It might not be too surprising to find that interventions that focus primarily on retrieving positive memories show short-term gains rather than longer-term effects. These interventions are unlikely to change the underlying memory representations, and instead may act primarily by providing the in-the-moment reward of the positive memory or potentially by increasing the accessibility of that positive memory (or related positive memories) for some period of time. However, as retrieval contexts shift (in time, in space, in brain-state), these influences might be expected to diminish in impact.

For longer-lasting influences to arise, it would seem essential for the underlying memory representation to be altered. Samide and Ritchey (2021) recently proposed that memory processes can be used as an emotion regulation device, helping to reframe the past and that the efficacy of these processes for emotion regulation may be tied to the completeness or strength of the recapitulation of the event in memory. This could even be one reason for the strong link between memory specificity and mental wellbeing; a specific memory has the opportunity to be updated in content and framing, while a general memory may not. This perspective also may shed light on the connections between overgeneral memory and depressive symptomology. An influential model of overgeneral memory proposes that it may be, in part, a cognitive avoidance strategy adopted to reduce negative affect (Williams et al., 2007), and a systematic review found evidence consistent with the idea that avoiding the retrieval of specific memories of an aversive event can reduce distress in the short-term (Sumner, 2012). Yet, over the long-term, this avoidance of specific memories appears to be harmful: Two meta-analyses have suggested that the presence of overgeneral memories at one time-point can predict greater depressive symptoms at follow-up (Sumner et al., 2010; Hallford et al., 2021; see also Chiu et al., 2019). By not retrieving specific memories, individuals may deprive themselves of opportunities to reframe the experience and to update the memory.

A recent study suggested that updating an underlying memory representation may be exactly what happens when, rather than asking people to focus on positive memories, people are asked to find positive meaning in a past negative event. Speer et al. (2021) found that participants who elaborated on the positive aspects of past negative events reported increased positive emotions and memory content upon future recollections of the same negative event, up to two months after the initial recollection. Paralleling these behavioral changes, the neural results suggested that the memory representations may have changed as a result of these reframings. During negative memory recollection, as memory content increased in positivity, neural activation patterns in regions associated with episodic memory retrieval (i.e., hippocampus) and reward-related processing (i.e., ventral striatum; Speer et al., 2014) became less similar to baseline activity profiles. Thus, by finding positive meaning in these past events, individuals may have changed the memory representations in ways that had long-term consequences. The authors proposed that the mechanisms may be akin to those engaged during positive reappraisal, consistent with the similarity between the neural activity engaged during positive meaning finding and that engaged in previous studies of positive reappraisal.

While more work is needed, there is reason to suspect that this type of positive reframing would be particularly powerful in its long-term consequences. In fact, a recent study found that memory-reframing helped children to remember a recent tonsillectomy more positively than those assigned to a control condition (Pavlova et al., 2022). It makes sense that changing the nature of the memory representation would have long-term consequences: In an animal model, artificially triggering a positive memory during the reactivation of a negative experience reversed the animal’s aversive behavior (Ramirez et al., 2015; Redondo et al., 2014), suggesting that reframing may be able to alter the memory representation. While this type of direct evidence does not yet exist, there is correlative evidence that older adults, who generally enjoy better mental wellbeing than younger adults even in the face of life stressors, are particularly good at this type of positive reframing. It is intriguing to consider whether there may be a causal link—whether part of the wisdom that is acquired with aging is the ability to positively reframe past negative experiences, and whether this tendency to reframe provides older adults with some of their resiliency (see Box 4).

Although we have so far focused on the benefits for mood, the power of positive memories extends into broader domains as well. Positive memories become integrally tied to our sense of self and become an important part of our life story (Berntsen et al., 2011; McAdams, 2001). Our ability to remember positive moments from our past is related to our self-esteem (Çili & Stopa, 2015). In this way, positive memories can be connected to our wellbeing by allowing us to maintain a positive self-concept.

Positive memories also serve important social functions (Rasmussen & Berntsen, 2009; Wolf & Demiray, 2019). We have already described how social context can enhance the value of memories (Speer & Delgado, 2020), with people willing to pay more to reminisce about socially relevant positive memories (a birthday party) rather than positive events that they experienced alone (receiving a good grade). Positive memories also can connect us socially; as Köhler et al. (2015) eloquently state, these memories constitute “the milestones of social communication” (p. 2). Reminiscing about past experiences can be a powerful way to improve positive affect (Bryant et al., 2005) and to solicit social support (Barry et al., 2019), and even is being explored as a way to boost cognitive function among older adults or those with dementia (Klever, 2013; Lazar et al., 2014). Thus, retrieving positive memories is rewarding in the moment and, through the social and integrative functions of reminiscence (Westerhof & Bohlmeijer, 2014) also can lead to other positive outcomes that can further potentiate those rewards.

Positive memories can propel us to help others. Children perform more good actions after remembering their past good actions (Tasimi & Young, 2016), and similarly, when adults remember specific instances when they have helped others in the past, this increases their prosocial intentions (Gaesser & Schacter, 2014). It may not even be necessary for people to remember their own good actions; specific memories for the good actions of others also may lead us to help. Ford, Gaesser, DiBiase, et al., (2018) found that individuals who remembered the details of others’ heroism during the 2013 Boston Marathon bombings were more likely to subsequently donate time or money to Boston-area charities while this helping behavior was lower in those who remembered fewer details of others’ heroism.

Using fMRI, Gaesser et al. (2019) found evidence that the way the medial temporal-lobe memory system and theory of mind networks were engaged related to this link to prosociality. Interestingly, when TMS was applied to a core node of the theory of mind network (the right temporal-parietal junction, RTPJ), there was no effect on the willingness to help, suggesting that it may be the episodic memory network engagement that plays the key role in the association to helping behavior. More work is needed to fully explicate how the retrieval of positive memories spur us toward prosocial intentions. But the extant data are exciting in suggesting that retrieval of positive memories may be beneficial not only to the rememberer but also to others through their prioritization of prosocial intentions.

## Implications of the Power of Emotional Memories

In this final section, we aim to plant some seeds for future directions of research that we think could grow from the literature we have reviewed. In some cases, there is promising research already underway. In others, to our knowledge, there is not yet much that is known, and so here we point out possible links for future research to investigate.

## The Power of Emotional Memories to Change our Moods

We have reviewed the power for positive emotional memories to be used as emotion regulation devices. Individuals tend to recall positive memories in order to counteract a negative mood (Joormann et al., 2007; Joormann & Siemer, 2004). More recent evidence has suggested this ability for positive memories to serve as emotion-regulation devices may come from the fact that they can serve as rewards (Speer et al., 2014), and buffer against the negative effects of stress (Speer & Delgado, 2017). We briefly discuss why positive memories, or positively reframed memories, may be particularly effective emotion regulation devices, and describe contexts in which their efficacy may be enhanced.

### Positive memory retrieval as an emotion regulation device

While there are many other emotion regulation strategies people can use (McRae & Gross, 2020), there may be specific benefits conveyed by the use of positive memories. For one, the use of positive memories to change one’s mood may require less training than other emotion regulation strategies. While individuals often have to practice extensively to become good at reframing and reappraising experiences, autobiographical memory retrieval is a common part of daily experience. Second, the use of positive memories may be implementable across a wider range of scenarios, including instances where the emotion is not elicited by any specific situation that can be reframed or instances in which it is a mood rather than a short-lived emotional reaction that must be regulated.

### Episodic specificity inductions to enhance negative memory reframing

It is not just positive memories that can provide positive impacts to our mental wellbeing. Negative memories can as well if we are able to reframe them so as to find the good that has come from them (Samide & Ritchey, 2021). In fact, reframing negative memories may be a particularly powerful way to boost our mental health, in long-lasting ways. As we described earlier, a barrier to such reframing may be if memories are retrieved in an overgeneral and semanticized way, rather than as an episodically rich memory. If true, then training individuals in how to retrieve specific memories may be advantageous.

Memory specificity training (Madore & Schacter, 2014; Raes et al., 2009), based on the cognitive interview (Geiselman et al., 1985), encourages individuals to thoroughly retrieve details of an event, using a guided process to enhance the retrieval of episodic details. There is promising work showing that specificity inductions can be linked to reductions in symptoms of PTSD (Moradi et al., 2014), depression (Neshat-Doost et al., 2013; Raes et al., 2009), and complicated grief (Maccallum & Bryant, 2011). It also may boost positive affect and decrease negative affect in healthy college students (Jing et al., 2016). Some of these benefits have been linked to the concept of *episodic reappraisal* (Jing et al., 2016) or the ability to reframe a negative experience that one is remembering or imagining.

### Power of reminiscence for grief

Autobiographical memory retrieval can be powerful for individuals who are grieving (Mroz & Bluck, 2019). Whether those memories are powerful in harming or helping depends on the way people use their memories. Wolf et al. (2021) revealed that after the loss of a loved one, individuals benefited from recalling autobiographical memories if they did so in ways that have been described as “self-positive” (Cappeliez & O’Rourke, 2006) – using memories to maintain identity, to problem-solve, and to prepare for one’s own death. They suggested that when used this way, past memories may help individuals to reframe their identity and their future without their loved one. To our knowledge, there have not been interventions focused on helping individuals to use their memories in these self-positive ways, but there may be promise for doing so as a way to help those who are grieving a loss.

## The Power of Emotional Memories in the Classroom

Educational psychology has been deeply influenced by research on executive functioning and cognitive control (Diamond & Lee, 2011). Yet the long-term memory literature has had relatively fewer intersections with the way classroom education is approached (Ofen, Yu, & Chen, 2016). There have been recent attempts to bridge this divide (Fandakova & Bunge, 2016), but to our knowledge there has been little discussion of how the literature on episodic emotional memories may be relevant to the classroom. We suggest the importance of considering two directions of connections.

### Emotional material may benefit from different study techniques

Students do not just learn about neutral content in the classroom. They read fiction and nonfiction written to trigger positive and negative emotional reactions, discuss current events that are emotionally charged, and study about diseases and treatments that may directly affect loved ones. The advice given for how to effectively study this information is almost entirely based on laboratory research using nonemotional stimuli.

There is reason to think that many of the effective-study principles developed through examination of memory for neutral content will extend to emotional content. For instance, high-quality sleep has been shown to benefit memory for emotional content at least as much as nonemotional content (Payne & Kensinger, 2018). But what about spaced rehearsal? Or emphasizing quizzing over re-studying? What about taking notes in visual form versus written or in ways that emphasize associations among concepts? Few of these study principles have been examined for emotional content. To the extent that the emotional enhancement of memory is reliant on similar processes as engaged for neutral material (*mediation model*), the benefits should remain similar. But it seems plausible that where the mechanisms begin to diverge, so might the most effective study strategies. For instance, in contrast to the robust “testing effect” advantage conveyed for neutral content, there have been mixed results for negative content, and some suggestion that rather than the broad benefits conveyed for nonemotional content, retrieval practice may benefit memory for associations with negative content (Jia et al., 2018) but not memory for the negative items themselves (Jia et al., 2019). It seems possible that the different patterns of accessibility for negative versus neutral memories may contribute to this disconnect: If negative content is associated with a prioritized search process, this may mean that its retrieval conveys fewer benefits when compared to the more-effortful search process engaged for neutral information. It also is plausible that retrieval practice primarily engages content linked to hippocampal-binding mechanisms and may be less efficacious for content linked to amygdala-binding mechanisms.

### Emotional memories and new learning

We have already described the power for emotional memories to at least transiently change our affective state. Classroom assignments are often designed to evoke these memories: In elementary school, children might be asked to write about a favorite experience or to consider when they have felt similarly to a protagonist. In high school and college, students may be encouraged to write about life experiences to gain facility in writing, or to connect their life experiences to material being presented. When this memory retrieval happens in the classroom, there are likely to be consequences for the processing of incoming information.

If emotional memories are changing individuals’ moods, this can then impact how they are processing incoming information. Individuals in a negative mood may attend to details and may process information in a more narrow, analytic fashion than people in positive or neutral moods (Clore et al., 2001). By contrast, participants in a positive mood are more likely to process information in a broader manner, focusing on the gist or global theme of the information, and often seeing creative connections among stimuli that others miss (Clore et al., 2001; Fredrickson, 2004). There is some work focused on customizing learning material based on real-time automated evaluations of students’ emotional states (Shen et al., 2009), although much of it has focused on students’ experiences of confusion, and so there remains much to be investigated.

In the classroom, it seems plausible that retrieval of emotional memories could be leveraged to encourage the relevant modes of information-processing. A student might be asked to think about a positive memory before engaging in a task requiring creative associations to be drawn. If positive memories can buffer from stress in educational contexts as they can in laboratory ones, there could be benefits to asking students to take a moment to retrieve a positive memory before handing out a pop-quiz or asking a student to read in front of the class.

## The Power of Emotional Memories within Digital Contexts

Memory theories are increasingly recognizing the role of fluctuating internal as well as external contexts in guiding retrieval outputs, and how emotional state interacts with these retrieval outputs (e.g., *eCMR model*). It has long been considered how the encoding-to-retrieval match in physical environments affects learning (Abernethy, 1940; Godden & Baddeley, 1975). With the advent of digital contexts, and their pervasive use during the COVID-19 pandemic, individuals may now be able to decide “where” they study or work—what virtual background or environment they use, and for what situations they use it.

There would be good reason to think that consistency of digital contexts across study and retrieval episodes would benefit memory. Indeed, Cox et al. (2021) found when events occurred in the same contexts, retrieval performance for those events was better compared with retrieval of events presented in different contexts. Thus, returning to the same “virtual boardroom” may help an individual to recall ideas previously discussed in that virtual context.

There is a potential downside, which relates to the simplicity of the digital contexts. The use of a virtual background for a video call is quite similar to the juxtaposition of a facial expression upon an unrelated scene. What happens if that face is of your boss who is critiquing your latest presentation? Will the negative affect from that interaction “bleed” onto the background, as can happen in laboratory settings (Palombo et al., 2021; Madan & Kensinger, 2021)? These may be important questions to address, so that individuals can understand when continuity of digital context is helpful because the context-match from one meeting to another allows for increased content recollection, and when the possible downsides of “affective bleed” predominate.

Another consideration for digital workspaces is whether staying within the same digital “space” may affect our ability to create event boundaries. Event segmentation theory argues that humans proscribe boundaries to life events to organize and optimize the mountain of information we encounter in everyday life (Kurby & Zacks, 2008; Zacks & Swallow, 2007). Importantly, event boundaries are thought to help protect emotional memories from interference, allowing important emotional memories to be protected and prioritized (Dunsmoor et al., 2018). Events are traditionally defined as a period of time at a specific location that has a beginning and an end (Kurby & Zacks, 2008). Studies in real world settings show memory for items is improved when they were presented across events instead of within events (Pettijohn et al., 2016; Smith, 1982; Smith & Rothkopf, 1984). Curious if spatial location plays the same role in a digital context, a recent study used virtual reality to systematically identify if spatial boundaries are necessary to improve memory performance (Logie & Donaldson, 2021). In a series of four experiments, researchers showed that removing spatial boundaries, including doorways, walls, and separate rooms from a virtual reality space did not negatively affect free recall memory performance. The authors found spatial events are not necessary to create boundaries and that temporal boundaries were sufficient to provide memory enhancements (Logie & Donaldson, 2021). Thus, learning or working within a digital space that does not involve changes in physical location (e.g., a student who attends different classes throughout the day sitting in the same place on the same computer) may still leverage event boundaries toward memory success in the same way as when we are interacting with physical environments. More needs to be done to examine whether this holds for emotional content being remembered.

The implications can extend beyond work, as digital spaces are increasingly being used for social connection purposes as well. In what ways do memories of a birthday party or memorial service differ when experienced online versus in person? How do any differences influence the power of those memories?

## Shifting the Balance of Hippocampal and Amygdala Binding Systems

In this review, we described how the hippocampus and amygdala may subserve different binding functions (Yonelinas & Ritchey, 2015) and may not always work synergistically (Bisby & Burgess, 2017). If indeed there can be shifts in the balance between the relative engagement of hippocampal and amygdala binding systems, this raises the question of whether there may be manipulations that can strategically shift these weights. Are there interventions that could increase the likelihood of remembering contextual details of high-arousal, negative experiences, by boosting the reliance on the hippocampal-binding system? Given that disruption of hippocampal mechanisms and the ability to remember specific, contextual details has been associated with depression (Belleau et al., 2019) and PTSD (Shin, 2006), this would seem an important question to examine. To our knowledge, there is no direct evidence to address this question, but we note two potentially promising directions for future research.

### Neurofeedback

It remains unclear how the balance of hippocampal and amygdala binding systems is determined. There is beginning to be promising evidence that neurofeedback can be used to downregulate amygdala activity (Brühl et al., 2014) and to enhance emotion regulation (see Linhartová et al., 2019 for review). For instance, Herwig et al. (2019) provided participants with neurofeedback of their own amygdala activity while they were instructed to use cognitive reappraisal. Over four weekly sessions, they noted significant reductions in amygdala activity. Interestingly, amygdala connectivity with the hippocampus also increased. They did not examine memory for the pictorial stimuli presented during the neurofeedback session, but this pattern raises the question of whether methods like this may be useful for achieving a greater balance between hippocampal and amygdala binding systems.

### Aerobic exercise

Extensive prior research has demonstrated that aerobic exercise is good for hippocampal function (Erickson et al., 2011) and may encourage cell growth in the hippocampus (Luo et al., 2019). Less is known about how exercise affects the amygdala. To date, most research has focused on the effect of exercise on emotion regulation, showing that exercise can boost prefrontal function (Ligeza et al., 2021) and can increase connectivity between the prefrontal cortex and amygdala in ways that have been interpreted as indicative of improved emotion regulation (Ge et al., 2021). If exercise is indeed increasing hippocampal function while decreasing amygdala engagement, it may create an intriguing scenario in which an emotional experience is remembered primarily with hippocampal-binding mechanisms engaged. To our knowledge, no work has examined whether participation in aerobic exercise programs or individual differences in aerobic fitness affect the types of details remembered about an emotional experience. Instead, the bulk of work has looked at how acute bursts of exercise around the time of learning or consolidation affect overall memory ability (Loprinzi et al., 2019 for review) or emotional memory ability specifically (Libkuman et al., 1999; Wade & Loprinzi, 2018). For example, future work could examine how exercise interventions affect associative as well as item memory for negative content.

## Conclusions

Our memories are a powerful tool with which we navigate our lives; we use our memories to remind us of our past, to make sense of our present, and to direct our future. The current review has highlighted the ways in which emotional valence enhances this power. Positive and negative emotion can make memories easier to retrieve, more richly reexperienced, and more likely to influence behavior. These changes are supported by a variety of mechanisms that guide how events are encoded, consolidated, retrieved, and altered over time. We argue it is important for the field of emotional memory to 1) gain a firm understanding of the similarities and differences between the characteristics and uses of negative and positive memories, 2) the mechanisms that support these differences, and 3) their implications in clinical, educational, and professional domains.
